# Experimental Investigation on Surface Quality Processed by Self-Excited Oscillation Pulsed Waterjet Peening

**DOI:** 10.3390/ma10090989

**Published:** 2017-08-25

**Authors:** Xiaolong Ding, Yong Kang, Deng Li, Xiaochuan Wang, Dongping Zeng

**Affiliations:** 1 Key Laboratory of Hydraulic Machinery Transients, Ministry of Education, Wuhan University, Wuhan 430072, China; xlding@whu.edu.cn (X.D.); 2008lee@whu.edu.cn (D.L.); xcw001@whu.edu.cn (X.W.); zengdongping@whu.edu.cn (D.Z.); 2 Hubei Key Laboratory of Waterjet Theory and New Technology, Wuhan University, Wuhan 430072, China; 3 School of Power and Mechanical Engineering, Wuhan University, Wuhan 430072, China; 4 Collaborative Innovation Center of Geospatial Technology, 129 Luoyu Road, Wuhan 430072, China

**Keywords:** SOPW, surface peening, surface topography, roughness, hardness, residual stress

## Abstract

High-speed waterjet peening technology has attracted a lot of interest and is now being widely studied due to its great ability to strengthen metal surfaces. In order to further improve the mechanical properties of metals, self-excited oscillation pulsed waterjets (SOPWs) were used for surface peening with an experimental investigation focused on the surface topography and properties. By impinging the aluminum alloy (5052) specimens with SOPWs issuing from an organ-pipe oscillation nozzle, the hardness and roughness at various inlet pressures and stand-off distances were measured and analyzed, as well as the residual stress. Under the condition of optimum stand-off distances, the microscopic appearances of peened specimens obtained by SEM were displayed and analyzed. Results show that self-excited oscillation pulsed waterjet peening (SOPWP) is capable of improving the surface quality. More specifically, compared with an untreated surface, the hardness and residual stress of the peened surfaces were increased by 61.69% and 148%, respectively. There exists an optimal stand-off distance and operating pressure for creating the highest surface quality. SOPWP can produce almost the same enhancement effect as shot peening and lead to a lower surface roughness. Although such an approach is empirical and qualitative in nature, this procedure also generated information of value in guiding future theoretical and experimental work on the application of SOPWP in the industry practice.

## 1. Introduction

Nowadays, the properties and work reliability of many metallic materials are in need of improvement with the development of scientific technology and industrialization processes, such as the precision instrument and aircraft industries [1]. The improvement of metal surface quality has attracted a lot of attention in the material optimization field. It is well-known that, surface roughness and surface integrity resulting from net-shape processing can largely determine the fatigue life and corrosion resistance of all kinds of metal materials [2,3,4]. Moreover, fatigue failure which generally develops on particular surface areas of engineering parts such as sharp corners, or welding points [5,6,7], is closely related to the surface qualities, such as surface roughness and residual stress. So, it is believed that surface treatment is a necessary procedure for better applications of metal materials.

Methods of physical treatment that can improve the quality of metal surface include shot peening (SP), laser peening (LSP), waterjet peening (WJP), and so on [8,9,10]. The positive effects of these methods on the surface properties are generally related to its ability to introduce a compressive residual stress state in the surface layer and to harden the surface by inhomogeneous plastic deformation caused by the shot flow, laser and waterjet [11]. However, each method has its shortcomings. For example, shot peening is destructive to the surface integrity which greatly determines the surface quality. When no protective laser-absorbent coating is used on the substrate, the laser shock processing causes a severe surface melting and vaporization, particularly for aluminum [12]. However, waterjet peening can avoid these two deficiencies. In order to improve the performance of waterjet peening, enough high pressure and kinetic energy are in need. As a result, ultra-high-pressure pumps and other reliable equipment are necessary, which greatly increases the costs and poses a huge challenge to the wide application of this technology.

As for waterjet peening, most researchers have concentrated on the theory and applications. Arola and McCain [13] first proposed the concept of waterjet peening for surface preparation of metal orthopedic implants by material removal, rather than by conventional deposition of porous coating, which results in little sacrifice in the material fatigue strength. They also did research on the surface strengthen of titanium and found that the parameters of waterjets could affect the surface properties. Sadasivam et al. [14] confirmed that elastic pre-stress had a significant effect on the magnitude of surface residual stress and the depth of compressive residual stress and had no effect on the surface texture. Barriuso et al. [15] performed an experiment to improve the surface of AISI 316 LVM and Ti6Al4V by waterjet peening without abrasive particles. They determined the reason for metallic piece volume loss and verified the viability and the application of pure waterjet peening in the field of surface strength. They conducted a number of experiments that paved the way for the practical application of waterjet peening.

In order to reduce energy consumption and improve the quality of surface peening, different forms of jets such as cavitation peening and abrasive waterjet peening have been created. Cavitation peening is a widely used method and has been investigated in the literature in recent years. For example, Soyama et al. [16] compared cavitation peening and shot peening for extending the fatigue life of metal materials. They found that the fatigue life of the shot-peened specimen was equal to or less than that of the machined-specimen, whereas cavitation peening extended the fatigue life. Also, Gao et al. [17] investigated the ultrasonic cavitation peening of stainless steel and nickel alloy. It was found that in the studied situations, ultrasonic cavitation peening can clearly enhance the surface hardness without significantly increasing the surface roughness. Ju et al. [18] investigated the microstructures in the near-surface layer of pure titanium by water cavitation peening. Besides, abrasive waterjet peening has also been applied in the process of peening, and researchers have made numerous investigations into the characteristics and surface qualities of peened surface. According to previous investigation, Sadasivam et al. [19] made an experiment to study the effects of elastic pre-stress and boundary in the process of abrasive waterjet peening. Results showed that the residual stress under pre-stress is up to 50% more than the original. Chen et al. [20] studied the surface strengthening properties of aluminum alloy with abrasive waterjet peening. Results indicated that abrasive waterjet peening creates a better surface topography than shot peening. Recently, the self-excited oscillation pulsed waterjet (SOPW) is gaining attention due to its unique characteristics which combine high instantaneous pressure and the cavitation effect.

SOPW is a new kind of efficient jet, which developed on the basis of the theory of transient flow, acoustic and fluid resonance. SOPW can produce great striking power due to its water hammer effect, which has a significant effect on the material surface strengthening [21]. The organ-pipe nozzle is a typical SOPW nozzle, which produces jets with higher pressure oscillation and larger separated vortex rings. Experiments show that under the same circumstances, the organ-pipe nozzle can produce jets of instant pressure 1.5–2.5 times higher than ordinary nozzles [22]. Due to these characteristics of SOPW, it is very suitable to be applied in the process of surface peening, with great advantages being generated. Additionally, the operating pressure needed for surface peening is lower than the continuous waterjet. Moreover, the surface roughness of abrasive-less waterjet peened metal may be lower than that of shot peening. This is because shot peening strengthens the surface by using the method of impacting with solid particles while abrasive-less waterjets uses pure water droplets. Note that the previous studies have been limited to continuous waterjet peening, cavitation peening and abrasive peening. Few studies have evaluated the influence of SOPW parameters on the material surface peening field.

The present paper describes an experimental investigation into the effects of SOPW parameters on surface peening with respect to mass loss, SEM observation, roughness, and surface hardness, as well as X-ray diffraction residual stress. It serves as a supplement to the waterjet peening technology so as to provide useful information for better applications of self-excited oscillation pulsed waterjet peening (SOPWP).

## 2. The Generation Mechanism of SOPWs

**Figure 1:**
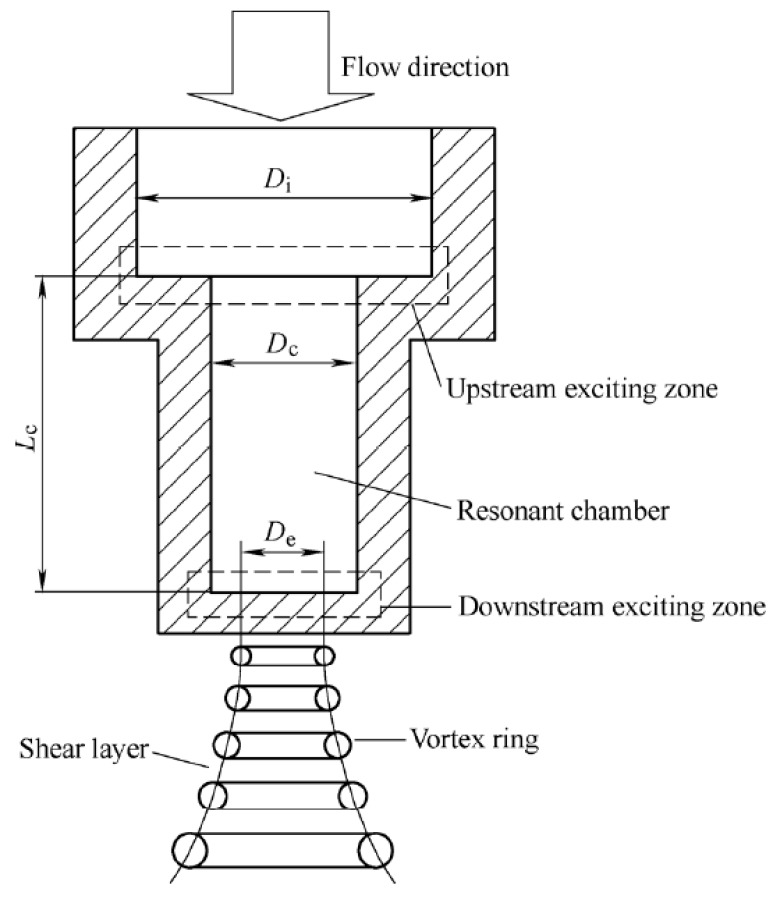
Schematic of configuration and operation principles of an organ-pipe nozzle.

In order to fully understand SOPWs’ generation mechanisms and characteristics, a preliminary analysis was carried out. As already established, several nozzles can effectively yield SOPWs but the most promising one is the organ-pipe nozzle. Figure 1 is a schematic diagram illustrating the configuration and working principles of an organ-pipe nozzle.

As is shown in Figure 1, a typical organ-pipe nozzle contains two exciting zones formed by two abrupt area contractions, and a resonant chamber between the contractions. Peak acoustic resonance can be achieved when a standing wave is formed in the chamber, whose diameter is D_c_ and length is L_c_. The standing wave is a result of the superposition of the pressure waves generated at the downstream area contraction and the waves reflected from the upstream area contraction, under the condition that the fundamental frequency of the nozzle is near the critical jet structuring frequency. The exact frequency for peak resonance is dependent on the end impedances, which in turn depend on the upstream contraction ratio (D_i_/D_c_) and the downstream contraction ratio (D_c_/D_e_), respectively [23]. According to the generation conditions of standing waves, it is known that if both D_i_/D_c_ and D_c_/D_e_ are large, then the first mode resonance in the chamber will occur when the sound wave length is approximately four times L_c_; while peak resonance, which has a wavelength of approximately two times L_c_, appears, if both D_i_/D_c_ and D_c_/D_e_ are close to one [24].

Once the standing wave is formed in the nozzle assembly, the natural tendency of an axisymmetric jet organizing into large structures will be greatly amplified. As a consequence, the pulsed waterjet is generated and the submerged jet shear layer organizes into large ring vortices emitting from the nozzle at a discrete frequency, and then cavitate to form toroidal bubbles called vortex rings, and a SOPW is successfully yielded.

As for surface peening, the water droplets with high instantaneous pressure hit on the material surface. The impact force is higher than a normal waterjet. The subsequently yield bubbles in the shear layer of the jet then collapse when impinging on a solid surface. It has been found that pressure of approximately 10 GPa and temperatures higher than 5000 k can be generated during the picoseconds of collapse of the cavitation bubbles, which is able to peen the surface of the material.

## 3. Experimental Setup and Procedures

### 3.1. Facilities and Setup

**Figure 2:**
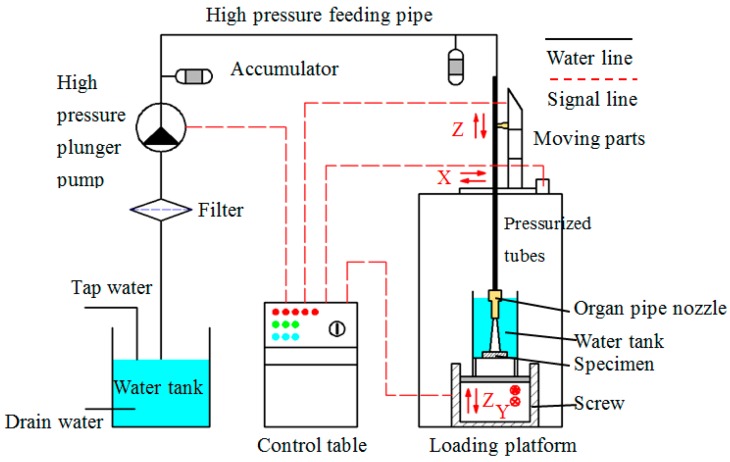
Schematic diagram of the experimental setup.

The experimental setup for the SOPWs peening tests is shown in Figure 2. The experiment was performed on a multifunction waterjet test bench, which was developed independently by our research team [25,26].

High pressure waterjets were provided from a motor-driven plunger pump whose maximum flow rate and pressure were 120 L/min and 60 MPa, respectively. The pump pressure, P could be continuously regulated though a control table that could change the working frequency of the motor. To attenuate the impact of pressure or flow rate fluctuations of the pump on the experimental results, two bladder accumulators were employed, with one positioned immediately near the pump and the other located as close as possible to the nozzle. Four pump pressures were used in the experiment, which were 15, 20, 25 and 30 MPa, respectively.

In each peening test, high pressure waterjet issued from the nozzle and impinged on a specimen located at the bottom of a vessel filled with water. The nozzle was mounted on a walking device that had X and Z motions with a precision of 0.1 mm, shown in Figure 2. In each test, after the stand-off distance was set, the position coordinates were recorded by the control system. In the pressurized stage, the nozzle was facing out of the specimen area. Consequently, the pump was started, and after the operating pressure stabilized at the designed value, the nozzle was then moved back to the recorded position with a maximum speed of 100 mm/s. Thus, the peening effect of impingement on the specimen on the way back could be neglected, as it happens in such a short time, and it certainly belongs to the incubation stage of the peening process, where the mass loss can be disregarded. In this way, the accuracy of the peening time in each test was ensured. Taking previous, related works on waterjet peening for reference and in association with our preliminary tests, during each peening test the specimen was exposed to the jet for 180 s to make the best surface quality. The stand-off distance, S, was defined as the distance from the nozzle exit to the surface of the specimen. During each test, stand-off distance was varied from 10 to 60 mm with an interval of 10 mm. The angle between the specimens and nozzle axis was 90°.

### 3.2. Nozzles and Specimens

A nozzle was designed according to the “Organ-pipe Design Manual” by Shen and Li [27] to give full play to the advantages of pulsed waterjets. The diameter of the inlet, chamber and exit were 13, 5 and 2 mm, respectively. The chamber length was calculated by using the following equation:

$$\frac{L_{c}}{D_{e}}=\frac{K_{n}}{M_{a}S_{d}\left(1+\beta \right)}$$

where $K_{n}=\{\begin{array}{c} \frac{2n-1}{4};\mathrm{for}\;\frac{D_{c}}{D_{e}}\gg \frac{1}{\sqrt{M_{a}}}\\ \frac{n}{2}\;;\mathrm{for}\;\frac{D_{c}}{D_{e}}\ll \frac{1}{\sqrt{M_{a}}} \end{array}\;n\;=\;1\;,2\;,3\ldots ,\;S_{d}$ is Strouhal number, $M_{a}$ is Mach number, $D_{e}$ is the nozzle exit diameter, $L_{c}$ is the chamber diameter, β is a correction factor.

**Figure 3:**
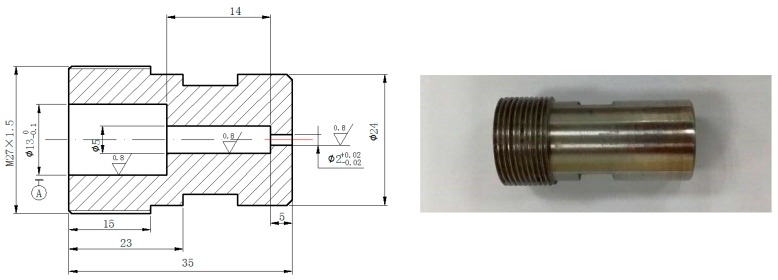
Profile and photo of the organ-pipe nozzle.

According to the calculation, the chamber length was 14 mm. and the exit length for the nozzle was 5 mm, which was determined by the strength of the nozzle material in order to make sure the nozzle would not be destroyed under experiment pressures. The profile and photo of the organ-pipe nozzle are shown in Figure 3.

For the specimen, 5052 aluminum alloy (Chinese Industry Standard) was selected as the test material. This aluminum alloy is commonly used in industry because of its excellent erosion resistance and medium intensity. The typical chemical composition and mechanical properties of 5052 aluminum alloy are given in Table 1 and Table 2, respectively. The 5052 aluminum alloy specimens were cut into cubes with a dimension of 100 mm × 100 mm × 15 mm. In addition, to ensure the accuracy and reliability of experimental results, the surface of the specimens were smoothed and film-coated during the production process through the rolling method. It had an average surface roughness, R_a_ of 0.5 μm. Therefore, no smoothing of surfaces was needed prior to the experiments.

### 3.3. Evaluation Methods

A precision electronical auto-balance with a maximum error of 0.001 g was used to measure the mass loss of specimens. The mass was calculated using the average of three measurements. All of the aluminum alloy specimens were dried prior to observation and measured by a dryer before and after the tests.

SEM was carried out by Tescan mira 3 (Brno, Southern Moravia, Czech Republic) to measure the surface topography of the specimens. According to the size of aluminum alloy crystals, 100 and 10 μm were chosen as the scanning accuracy in our tests. By comparing two different images, the changes of surface topography between lower pressure and higher pressure could be analyzed. Then, the principles of the SOPWP were also determined.

The roughness of the aluminum alloy was measured by an optical profiler which is specially manufactured for our laboratories. It was able to measure to the depth of 1000 μm with an optical sensor APM1. The area of 2 mm × 2 mm was gauged by taking one measurement point every 2 μm. The 3D surface profiles were shown as the comparison and acknowledgement of the SEM. Besides, cross section contours were also plotted to give a direct view of surface undulation and to calculate the roughness of the peened surface. The arithmetical mean deviation of the profiles (R_a_) and maximum height of the profiles (R_z_) were plotted against the stand-off distance at four operating pressures. The uncertainty of surface roughness measurement mainly came from the interference phase measurement error and contour calculation error with the application of the optical profiler. According to the equipment guide and our specific experimental conditions, the uncertainty of surface roughness measurement is less than 5%.

The hardness of the specimens was tested with a fully functional micro-Vickers hardness tester MC010-HVST-1000Z (Shanghai HiTop Tech-Development Co., Ltd., Shanghai, China). Due to the hardened layer depth of the specimens, the processed specimens were measured with the applied load of 9.807 N and the indenter movement time of 3 s. As a result, the speed of the indenter was kept at a level of 0.1 mm/s. The maintained time of the applied load was 15 s. The indentation depths were about 5–10 μm. The errors of the hardness measurements mainly came from the tester itself and specimen’s surface quality. In order to eliminate the experimental uncertainty, the specimens were measured ten times in different places on the peened surface. Thus, accurate data for the surface hardness was the average of ten measurements in order to make the results reliable. The error of the surface hardness measurement is less than 3%.

To obtain quantitative values for the residual stresses on the material surface, X-ray diffraction (XRD) measurements were carried out by means of an X-Stress 3000 StressTech device (American Stress Technologies, Inc., Vaajakoski, Finland). The residual stress was measured by d-sin 2ψ method and it can be described as:

$$\sigma =-\frac{E}{2\left(1+V\right)}{\cot\;\theta }_{0}\frac{\pi }{180}\frac{\partial \left(2^{\theta }\right)}{\partial \left({\sin\;}^{2}\psi \right)}$$

where E is the Modulus of elasticity and V is Poisson’s ratio. $\theta_{0}$ is the diffraction angle when the incident angle is 0. We define K = $-\frac{E}{2\left(1\;+\;V\right)}\cot\;\theta \frac{\pi }{180}$, and $M=\frac{\partial \left(2^{\theta }\right)}{\partial \left({\sin\;}^{2}\psi \right)}$. The above formula can be written as:

σ=K·M

where K is elastic constants and M is the slope of $2^{\theta }$ and ${\sin\;}^{2}\psi$. The elastic constant of the aluminum alloy specimen is 674.82 by the calculation above. M can be measured by the XRD. The XRD device has Cr radiation Kα, irradiated area of 1 mm^2^. The tube voltage of the device is 30 KV and the tube current is 9 mA. The exposure time is 30 s. In the experiment, the diffraction angles (2θ) were scanned at 6 different ψ angles ranging from −45° to 45°. With the change of angles, the diffraction angles are around 138°, and the XRD peak is about 80. Due to the curvature of the fatigue specimen the measurements were executed in the 90°direction while it was not possible to measure other stress components to determine the complete stress tensor and the principal stresses. The residual stresses of normal direction were measured to analyze the SOPWP effect on the surface strengthening. The error of XRD mainly comes from 6 aspects which are calculation error, sample selection error, device measurement error, manual operation error and the status of the material surface. In this experiment, the last three aspects were the main factors. Taking these three factors into consideration, the error of surface residual stress measurement is less than 3%.

## 4. Results and Discussion

### 4.1. Mass Loss

Mass loss is an important indicator to ensure the feasibility of surface peening. If the parts weight decreases significantly in the surface treatment process, it will not only affect the physical properties of the material itself, but also adversely affect metal parts assembly and operation.

**Figure 4:**
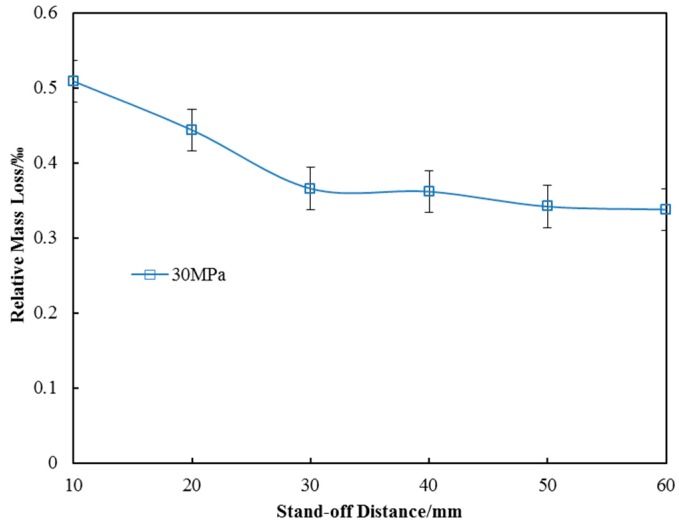
The mass loss of the specimens.

As is well-known, the higher the pressure, the greater the impact force of the waterjet beam. In order to verify the maximum mass loss under all operating pressures, the pressure of 30 MPa was selected for this test. Figure 4 shows the relative mass loss of the specimens in the process of SOPWP. It is clearly shown that the mass loss decreases as the stand-off distance increases. Moreover, the slope of the mass loss curve is decreases with the increase in stand-off distance, indicating that the impact capacity of the waterjet beam is weakened. When the operating pressure is 30 MPa and the stand-off distance is 10 mm, the relative mass loss reaches its maximum value of 0.5‰. This is an acceptable value in the surface peening process.

There are two main reasons for the specimen mass loss in the process of SOPWP. Firstly, water hammer effect was generated due to the unique characteristics of SOPW itself. To be more specific, according to the theory of water hammer effect, the fluctuations in the instantaneous flow produce a huge instantaneous pressure which is 1.5–2.5 times that of a continuous waterjet. When high- speed water droplets impact on the aluminum alloy surface, most of the surface crystals bear the impact of the water droplets, and then generate plastic deformation. A small amount of metal crystals are eroded and forced to leave the specimens surface. Secondly, as all the experiments were conducted in a submerged environment, high-speed gradient was generated on the boundary between the high-velocity fluid and the surrounding stationary fluid. The regions where the local pressure was below the local vapor pressure appeared in turn, to stimulate the growth of cavitation nuclei. The cavitation bubbles grew further by evolving in the jets until they were close to the surface. In the process of bubble breakup, the high local pressure and micro jets impacted on the peening surface. As a result, the metal crystals were removed and the mass was slightly smaller than the untreated one.

### 4.2. Surface Topography

**Figure 5:**
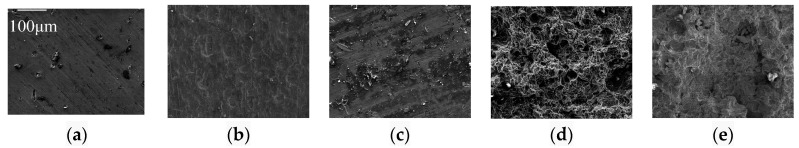
SEM of the peened specimen’s surface at the scale of 100 μm (**a**) untreated; (**b**) 15 MPa; (**c**) 20 MPa; (**d**) 25 MPa; (**e**) 30 MPa.

**Figure 6:**
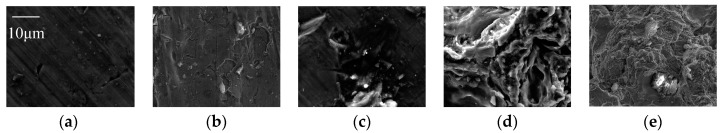
SEM of the peened specimen’s surface at the scale of 10 μm (**a**) untreated; (**b**) 15 MPa; (**c**) 20 MPa; (**d**) 25 MPa; (**e**) 30 MPa.

SEM was performed to analyze the microstructure of the specimens. These test results can help us to observe the peened surface micro topography directly, and then analyze the principles of the SOPWP. Figure 5 and Figure 6 are the surface topography of the processed aluminum alloy specimens. Figure 5 shows the surface topography which was treated by SOPW using the operating pressures of 15, 20, 25 and 30 MPa at the scale of 100 μm, as well as an untreated one. Figure 6 shows an untreated surface and the peened specimen’s surface treated by SOPW using the same pressures at the scale of 10 μm.

As is seen from Figure 5a, the topography of the specimen surface is relatively smooth, which indicates that the original surface roughness is low enough to perform the tests. However, Figure 5b–e show different surface topographies. For example, in Figure 5b, there exist a few concave points on the surface. The concave points are dense but not too deep, indicating that the waterjet beam is capable of generating impact force on the metal surface. However, because the pressure is 15 MPa, the water droplets do not have enough energy to cause serious deformation. More details were showed in Figure 6b. There are a few small particles attached on the specimen surface which are generated by the waterjet impaction. The diameters are about 2–5 μm. The concave points are also found at the scale of 10 μm and the depths are no more than 1 μm. Generally speaking, with an increase in operating pressures, surface topography becomes rougher according to the next images. Finally, in Figure 5e when the operating pressure is 30 MPa, the specimen surface experiences a severe plastic deformation. As we can see, the peened surface has deep pits and a number of folds. Lots of small particles have appeared and gathered in the pits inside. On a smaller scale, Figure 6e gives a more detailed and clear visual illustration of the pits and folds. The diameters of these pits vary from each other. Among the figures, Figure 5d,e show similar characteristics of surface topography. This illustrates that the SOPW peening ability is almost the same when the operating pressures are 25 and 30 MPa. This kind of phenomenon also affects the surface roughness, hardness and residual stress in this experiment. The specific reasons are analyzed in the following section. Although the micro pits have adverse effects on material surface quality, a comprehensive analysis needs to be conducted to determine the optimal parameters.

When SOPW hits the specimen surface, there are two factors that affect the surface topography. On one hand, high-speed water droplets have a strong ability to compress and erode. Due to the characteristics of SOPW, the droplets hit the surface with a certain frequency with high pressure. The surface metal crystals do not have enough compressive stress. Thus, severe plastic deformation occurs on the specimen surface. As a result, the surface becomes rugged. The specimen surface crystals are compressed, fractured and deformed. On the other hand, as mentioned above, the generation of cavitation also plays an active role during the process of peening. The high temperature and pressure generated by the collapse of the bubbles can also affect the surface topography. The tiny particles may be caused by micro waterjets during cavitation. As a result, the SOPW has the ability to strengthen the material surface by generating the plastic deformation and thus, we know that with the increase in the waterjet pressure, the specimen surface gets rougher.

### 4.3. Surface Roughness

In order to further explore the relationship between surface roughness and SOPW parameters, the surface roughness measurements were conducted with an optical profiler. The 3D view, surface profiles and roughness distribution curve were shown to analyze the influence of SOPW on surface roughness.

**Figure 7:**
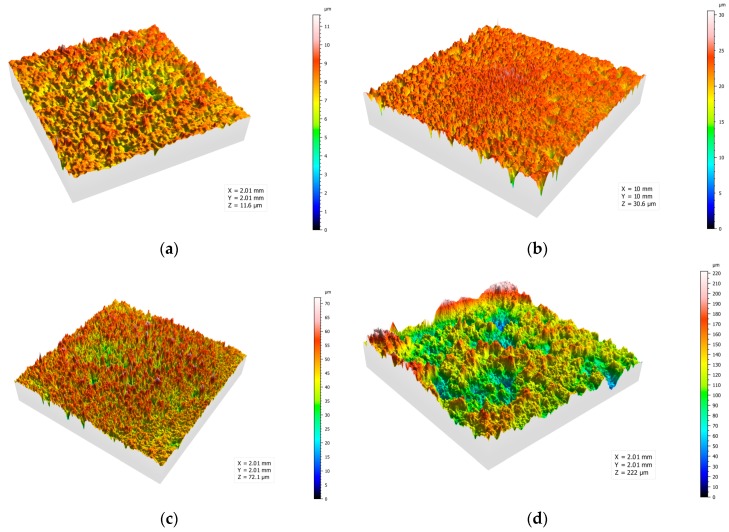
3D view of the surface topography at the operating pressures of (**a**) 15 MPa; (**b**) 20 MPa; (**c**) 25 MPa; (**d**) 30 MPa.

Figure 7a–d show the 3D view of the surface topography from the optical profiler at 15, 20, 25 and 30 MPa. The stand-off distance is optimal at the parameter of 20 mm. As a whole, the specimen surfaces are rough with concave points and compressional folds. The height of the concave points ranges from 8 to 160 μm. The surface roughness increases with the increase in operating pressure, and the depth and height of the concave points become greater from an intuitive point of view. Especially in Figure 7d, the concave points are overly impacted to form larger pits. We can infer from these images that concave points are formed by the impacting of SOPW, and the droplets cause metal crystals to compress, bend and deform. These crystals behaviors transform the mechanical property of the specimen surface.

**Figure 8:**
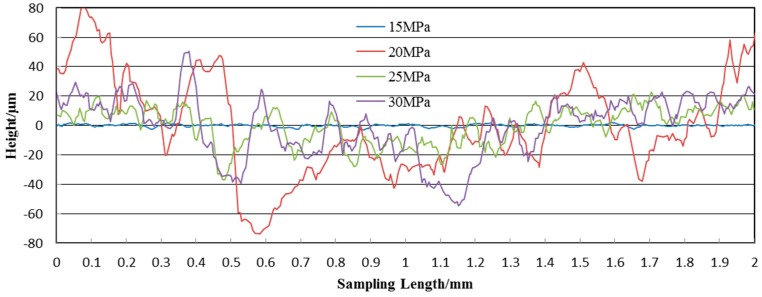
Profile line of the peening specimen surface at different operating pressures.

Figure 8 shows the profile lines of the peened specimen surfaces at four pressures. As we can see, the peak and valley values of the curves increase with the increasing pressure. For instance, the peak values are 15 and 80 μm when the pressures are 15 and 30 MPa, respectively. Besides, the trend of the profile lines is almost the same in Figure 8 at 20, 25 and 30 MPa. There is only a mild increase in the surface roughness of the specimen. In contrast, the profile line under the pressure of 15 MPa exhibits significant changes in the curve patterns. It has more ups and downs than the others. This can be explained by the energy density and intensity in the water droplets. When the pressure is 15 MPa, each drop does not have the ability to destroy the surface alone. Although a large number of water droplets hit the surface continuously, they can only produce shallow indentations. However, as the pressure increases, water droplets have the energy to erode the surface. Each drop has a slight compression effect on the surface. After a long period of accumulation, the surface of the specimen has large potholes. This is just a qualitative analysis, quantitative analysis will be carried out next.

**Figure 9:**
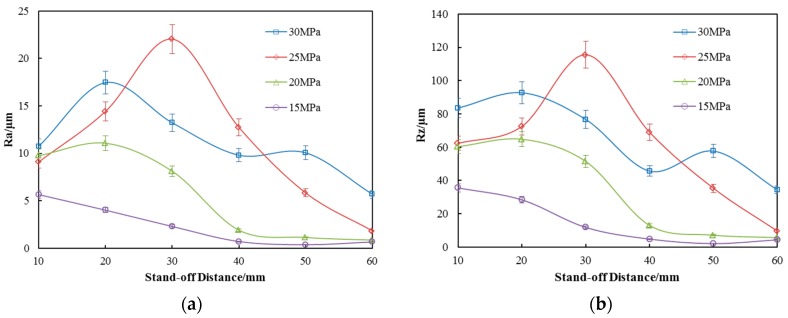
Roughness distribution of the specimens’ surface (**a**) R_a_; (**b**) R_z_.

The arithmetical mean deviation of the profile (R_a_) and maximum height of the profile (R_z_) against stand-off distance under the four operating pressures are plotted in Figure 9a,b, respectively. The R_a_ of the untreated specimen surface is 0.5 μm which can be used as a baseline for comparison.

It is obvious that the SOPWP can make the surface rougher than the untreated surface at almost all the test stand-off distances; meaning, the peening capabilities of SOPWs are strong. This is in agreement with the previous findings on surface topography, which indicates the reliability of this experiment to a certain degree. Moreover, by taking the mass loss (shown in Figure 4) into consideration, it can be determined that the mass loss caused by the peening process can be disregarded in the following discussion.

Figure 9a shows the arithmetical mean deviation of the profile (R_a_) against stand-off distance under the four operating pressures. R_a_ is arithmetic mean value of the absolute value of the contour height. It is clearly observed in Figure 9a that, the R_a_ increases with the increase in operating pressure. The SOPWP has a specific stand-off distance where the R_a_ reaches a maximum. The R_a_ first goes up and then drops with the increase in stand-off distance, which is independent of the operating pressure. However, the specific stand-off distance for each pressure is a little different. The existence of a specific stand-off distance depends on two facts. First, the organ-pipe nozzle produces different waterjet oscillation effects under different operating pressures. Secondly, the cavitation bubbles generated around the periphery of the jet need a certain time to grow to the appropriate size, until they collapse and yield micro jets or pressure strong enough to strengthen the surface. In more specific terms, when stand-off distance is 10 mm and the operating pressure is 30 MPa, the R_a_ is about 10.8 ± 0.54 μm. Then, the R_a_ increases with the stand-off distance increasing until the stand-off distance reaches 20 mm. The R_a_ reaches a maximum value of 17 ± 0.85 μm. Then, the R_a_ in all operating pressures decreases with the increase in stand-off distance except for an operating pressure of 25 MPa. When the pressure reaches 25 MPa and the stand-off distance is 30 mm, the surface average roughness (R_a_) reaches its maximum value, 22 ± 1.1 μm which is much larger than the one at 30 MPa. Lastly, the R_a_ of all the pressures goes down when stand-off distance is greater than 30 mm.

Figure 9b shows the maximum height of the profile (R_z_). It is obvious that the tendency of R_z_ against stand-off distance under the four operating pressures is similar to R_a_. There is just a numerical difference. The R_z_ first goes up and then drops down with the increase in stand-off distance, which is independent of the operating pressures. At the stand-off distance of 20 mm, the R_z_ reaches a maximum of 95 ± 4.75 μm with a pressure of 30 MPa, and the R_z_ reaches a maximum value of 120 ± 6 μm when the operating pressure is 25 MPa. Lastly, the R_z_ of all the pressures goes down when the stand-off distance is greater than 30 mm. It can be inferred that the maximum height of the profile shares the same characteristics as the arithmetical mean deviation of the profile.

As for the trend of surface roughness described above, it was interesting to find that the roughness at 25 MPa is different with respect to the others. At the stand-off distance of 30 mm and 40 mm, the roughness of the 25 MPa is greater than the others, especially at 30 MPa. Combined with the above description of the surface topography, there are several ways to explain this phenomenon. First of all, the impact effect of SOPW is related to operating pressures. It is not a simple linear relationship. There exists an optimal pressure that can make the oscillation effect most obvious. The oscillation intensity can have a significant effect on surface topography and surface hardness. Secondly, according to the generation mechanism of SOPW, the exact frequency for peak resonance is dependent on the end impedances, which in turn depend on the upstream contraction ratio (Di/Dc) and the downstream contraction ratio (Dc/De), respectively. In other words, the specific geometric parameters and operating pressure determine the SOPW behaviors. In our experiment, when operating pressure is 25 MPa, the nozzle has the best impinging performance when compared to other pressures. So, the surface hardness at a pressure of 25 MPa is higher than that at a pressure of 30 MPa.

In general, the results clearly show that the SOPWP has an obvious effect on the surface roughness at different operating pressures. Surface roughness is an important evaluation indicator in the manufacturing process related to mechanical coordination, resistance to corrosion and abrasive resistance. It is one of the most significant indicators that determine the part’s qualities. As we carried out the SOPWP to strengthen the surface, roughness is something that had to be suppressed. The experiments results show that the roughness has improved somewhat. However, compared to shot peening and abrasive waterjet peening where roughness is more than 30 μm, the SOPWP surface roughness is much less and smoother. It is also helpful in the following processing procedure.

### 4.4. Surface Hardness Distribution

As severe plastic deformation is generated in the process of SOPWP, the surfaces roughness is increased to a certain degree which is negative to the surface property [28]. However, the surface metal crystals are squeezed due to the plastic deformation, resulting in the formation of a hardened layer. In order to determine the surface hardness variation, Vickers hardness tests were conducted with different operating pressures and stand-off distances.

**Figure 10:**
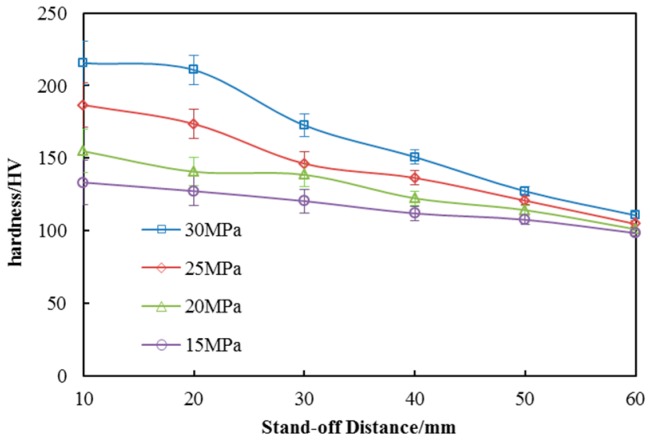
Vickers hardness distribution of the surfaces.

The surface hardness distribution is plotted against stand-off distance under four operating pressures in Figure 10. As we can see from the graph, the surface hardness of peened specimens is actually greater than that of untreated specimens. In addition, the Vickers hardness is increasing with the increase in operating pressure. For example, when the stand-off distance is 10 mm, the Vickers hardness of the surfaces are 215.45 ± 6.46, 186.6 ± 5.6, 154.8 ± 4.64 and 133.25 ± 4 HV at the operating pressures of 30, 25, 20 and 15 MPa, respectively. The average of treated-surface hardness is 1.5 times greater than the untreated surface. This is equivalent to the shot peening and better than conventional waterjet peening. Besides, the surface hardness decreases with the increase in the stand-off distance which is independent of operating pressure. The downward trend is basically linear. This indicates that the instantaneous pressure of SOPW goes down with the increase in stand-off distance. According to the theory of waterjets, the geometry of the jet is divided into the initial region, the transition region, and the basic region. The velocity of the core region in the initial region is the same as the velocity of the nozzle outlet which is greater than other regions. The length of the initial region is decreased with the decreasing pressure. As a result, when operating pressure is 30 MPa, the hardness is almost the same as during the stand-off distances from 10 to 20 mm, which is in the core region of the initial region. When the stand-off distance is outside the core region, the jet velocity and the pressure decreases with the increase in stand-off distance.

Surface hardness is a measuring method for solid matter surface resistant to various kinds of permanent shape change when a compressive force is applied. It relates to wear resistance, pressure resistance and stability of material surface. Generally speaking, surface hardness is an important indicator of the normal use of metal materials. SOPWP improves the surface hardness of the material by the cold-processing method. Due to the characteristics of SOPW, the instantaneous pressure is much larger than that of the continuous waterjet. The droplets with more kinetic energy impact on the material surface directly, causing severe plastic deformation. As a result, the hard layer is deformed. In the process of peening, the metal crystals are compressed by water droplets. The original arrangement of metal crystals is damaged and the crystals are forced to move away from the original location. Crystal dislocation and folds are thus generated. This dislocation and the folds are used to protect the internal metal from friction and extrusion. Besides, the hardened layer is beneficial to prevent the crack initiation from the metal surface. So, the SOPWP achieves the same goal with a smaller pressure than conventional waterjet peening.

### 4.5. Surface Residual Stress Distribution

In the process of manufacturing, the stress induced by cutting or local temperature will remain on the surface. Generally speaking, these stresses are almost residual tensile stress which largely contributes cracks [29,30]. In other words, the fatigue life of metal materials will be greatly improved if the residual tensile stress becomes smaller or translates into residual compressive stress. Therefore, the SOPWP process aims at generating residual compressive stress on the surface. The untreated surface residual stress is about −36.2 MPa as it was produced by the rolling method.

**Figure 11:**
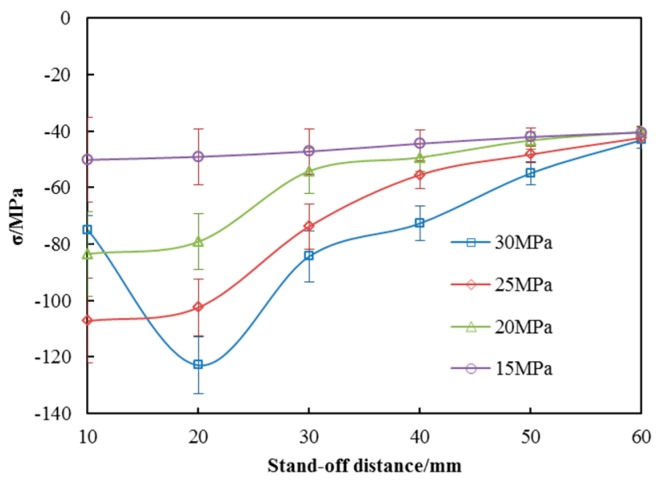
Residual stress distribution of the peened surfaces.

Figure 11 shows the residual stress distribution of the peened surface against stand-off distance at four operating pressures. As we can see from the graph, residual compressive stresses were indeed produced as the stresses are negative along the vertical coordinates. As the stress of untreated surface is −36.2 ± 1.81 MPa, the maximum of the stress value by SOPWP is −122.35 ± 6.12 MPa indicating that the SOPWP is able to increase the residual compressive stress. Furthermore, the residual compressive stress is slightly decreased when the stand-off distance increases. This is because the surge pressure goes down when the nozzle gets far away from the specimens. The residual compressive stresses are increased with the increase in the operating pressure, in general. However, when the operating pressure is 30 MPa and the stand-off distance is 10 mm, the value of the residual stress is smaller. By observing the surface topography, it is found that the surface which peened at 30 MPa and 10 mm was slightly eroded and the most superficial metal was gone in the process of peening. It can be inferred from this phenomenon, that at the beginning of the peening process, the hardened layer was generated and the residual compressive stress was accumulated by the water droplet impact. With the extension of the SOPWP time, the power is so great that the surface is not strong enough to resist impacting. As a result, the hardened layer generated by water droplets impacting was eroded off. The residual compressive stress does not have enough time to accumulate under such circumstance.

The residual compressive stresses are obviously improved by SOPWP according to the results above. It can be argued that the deformation energy is stored in the material by the SOPWP application. A material having compressive residual stress helps to prevent brittle fracture because the initial crack can be formed under compressive (negative tensile) stress. To cause brittle fracture by crack propagation of the initial crack, the external tensile stress must overcome the compressive residual stress before the crack tips experience sufficient tensile stress to propagate. So, it can be confirmed that the larger the residual compressive stress, the longer the fatigue life. Under the experimental conditions, the highest residual compressive stress occurs at an operating pressure of 30 MPa and stand-off distance of 20 mm. Under such parameters, R_a_ and R_z_ of the specimen surface are 17 and 86.2 μm, and the hardness of the specimen surface is 212 HV. Taking the roughness and hardness, as well as the residual stress of the specimen surface into consideration, the optimal parameters can be determined as 30 MPa and 20 mm. With the obtained experimental parameters, the physical properties of the peened materials can be applied in industrial practice.

## 5. Conclusions

In this paper, we performed an experimental investigation into the surface peening by SOPW and how it is influenced by operating pressure and stand-off distance. Unfortunately, there is currently little literature on the pulsed waterjet peening process, and these discussions are somewhat speculative. However, this is a novel method for peening surfaces with less roughness than generated by shot peening and it may also help improve the properties of alloy surfaces. The main conclusions of the study are as follows:

(1) SOPWPs are capable of improving the surface quality. Only with proper waterjet parameters, favorable hardness and residual stress as well as hardness can this be obtained. By comparing the SEM results, one can have a preliminary and visual understanding of the effects of waterjet parameters on surface peening topography.
(2) Compared with an untreated surface, the SOPWP surface increased its surface hardness and residual stress by 61.69% and 148%, respectively. Operating pressures and stand-off distances have a great effect on the peened surface quality.
(3) By evaluating the aggregative indicators, there exists optimal parameters to strengthen the surface properties in this experiment. At an operating pressure of 25 MPa and stand-off distance of 20 mm, the surface quality is better than the others.
(4) Although the surface roughness is increased in the process of SOPWP, it generates lower surface roughness than shot peening. That indicates that SOPWP can produce the same enhancement effect compared with shot peening with a better surface quality.

## Figures and Tables

**Table 1:** Chemical composition of the 5052 aluminum alloy (wt %).

Al	Mg	Cu	Zn	Mn	Cr	Fe	Si	Ti
Remain	2.2–2.8	≤0.1	≤0.1	≤0.1	0.15–0.35	≤0.4	0.07	0.0065

**Table 2:** Mechanical properties of the 5052 aluminum alloy.

Tensile Strength (MPa)	Offset Yield Strength (MPa)	Modulus of Elasticity (GPa)	Annealing Temperature (°C)
170–305	65	69.3–70.9	345
